# Conformational flexibility of a free and TCR-bound pMHC-I protein investigated by long-term molecular dynamics simulations

**DOI:** 10.1186/s12865-022-00510-7

**Published:** 2022-07-29

**Authors:** Lisa Tomasiak, Rudolf Karch, Wolfgang Schreiner

**Affiliations:** Institute of Biosimulation and Bioinformatics, Center for Medical Statistics, Informatics and Intelligent Systems, Medical University of Vienna, Spitalgasse 23, 1090 Vienna, Austria

**Keywords:** Molecular dynamics, Major histocompatibility complex, T cell receptor

## Abstract

**Background:**

Major histocompatibility complexes (MHCs) play a crucial role in the cell-mediated adaptive immune response as they present antigenic peptides (p) which are recognized by host T cells through a complex formation of the T cell receptor (TCR) with pMHC. In the present study, we report on changes in conformational flexibility within a pMHC molecule upon TCR binding by looking at molecular dynamics (MD) simulations of the free and the TCR-bound pMHC-I protein of the LC13-HLA-B*44:05-pEEYLQAFTY complex.

**Results:**

We performed long-term MD simulations with a total simulation time of 8 µs, employing 10 independent 400 ns replicas for the free and the TCR-bound pMHC system. Upon TCR ligation, we observed a reduced dynamic flexibility in the central residues of the peptide and the MHC α1-helix, altered occurrences of hydrogen bonds between the peptide and the MHC, a reduced conformational entropy of the peptide-binding groove, as well as a decreased solvent accessible surface area.

**Conclusions:**

In summary, our results from 8 µs MD simulations indicate a restricted conformational space of the MHC peptide-binding groove upon TCR ligation and suggest a minimum simulation time of approximately 100 ns for biomolecules of comparable complexity to draw meaningful conclusions. Given the relatively long total simulation time, our results contribute to a more detailed view on conformational flexibility properties of the investigated free and TCR-bound pMHC-I system.

**Supplementary Information:**

The online version contains supplementary material available at 10.1186/s12865-022-00510-7.

## Background

Major histocompatibility complex type I (MHC-I) or II (MHC-II) molecules present antigenic peptides (p) which are recognized by the host T cells and hence play a crucial role in the cell-mediated adaptive immune response. The T cell activation and the subsequent immune reaction are initiated by the T cell receptor (TCR) forming a complex with the peptide-presenting MHC (pMHC) [1]. MHC-I molecules are membrane bound proteins consisting of three α-domains (α1, α2, α3) and a ß2-microglobulin, where α1 and α2 form the peptide-binding groove with two α-helices on the sides and eight ß-strands on the floor [2, 3].

While crystallographic data have played a considerable role in increasing our understanding of the structural basis of TCR-pMHC engagement, they only provide a static picture and do not give comprehensive insight into the inter- and intra-molecular dynamics. In a recent study by Fodor et al. [4], the shortcomings of exclusively looking at static pictures of biomolecules were emphasized through uncovering significant conformational plasticity of human pMHC-I systems by crystallographic ensemble refinement techniques and molecular dynamics simulations. Their findings indicate that structural differences between pMHC and TCR-pMHC conformations might not necessarily be due to the binding process itself, which was proposed by former studies looking at static structures [5], but rather due to intrinsic flexibility. As the interest in the dynamics of immune response related molecules grew [6], the structure and dynamics of the *peptide* have generally been the focus of both experimental and computational studies. In a recent review, Ayres et al. [7] concluded that the mobility of peptides within MHC binding grooves impacts TCR binding, influencing association rates and entropic penalties. Peptides with higher mobility are therefore recognized more weakly by TCRs, with correspondingly reduced antigenicity. But these authors also discussed the fact that peptides can influence the motions of the MHC peptide-binding groove, especially of the α1- and α2-helix [8–10]. The focus on conformational dynamics of the MHC has grown in the past years and effects on TCR recognition and associated disease susceptibility has received great attention [11–13].

Molecular dynamics (MD) simulations have been used to provide information that goes beyond static structures and to give insight into the dynamic behavior of the TCR-pMHC complex. Narzi et al. [14] used 400 ns MD simulations to study two human MHC-I subtypes, HLA-B ∗ 27:05 and HLA-B ∗ 27:09, presenting a viral and three self-peptides. They could show that differences in the dynamic behavior, such as increased flexibility of the α1-helix and/or an opening of the binding groove of HLA-B ∗ 27:05 compared to HLA-B ∗ 27:09, are caused by very small structural differences in MHC subtypes (only one amino acid exchange in position 116). Gur et al. [15] conducted a similar study looking at HLA-B51 and HLA-B52 which only differ by two amino acids located in the B pocket [16] of the antigen-binding groove, while only HLA-B51 is associated with Behçet’s disease. Using all-atom conventional MD simulations of 4.8 µs in total length, these authors investigated the dynamics of the two HLA alleles bound to three different peptides and found that in HLA-B51 all peptides fluctuated to a larger extent due to looser binding compared to HLA-B52. Knapp and Deane [17] investigated the free and LC13 TCR-bound HLA-B*08:01 in complex with the Epstein-Barr viral peptide and 172 single-point mutations of the peptide using 100 ns MD simulations. In the bound state, these authors reported more hydrogen bonds (H-bonds) between the peptide and the MHC as well as altered flexibility patterns in the MHC helices and the peptide.

**Fig. 1:**
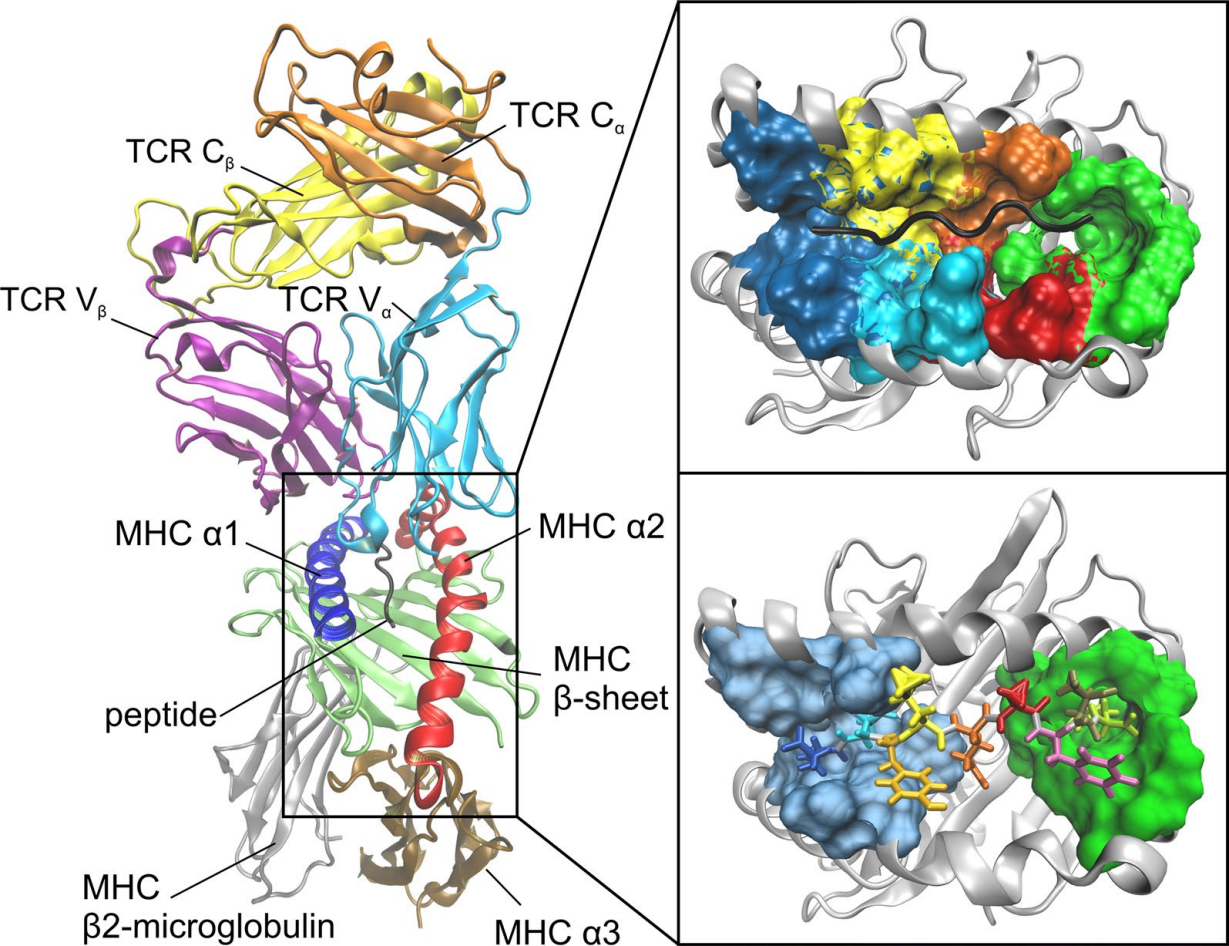
Left panel: Cartoon representation of the pMHC-TCR complex from the crystal structure 3KPS with domains MHC α1 (blue), MHC α2 (red), MHC α3 (ochre), MHC β-sheet (lime), MHC β2-microglobulin (silver), peptide (black), TCR constant domain C_α_ (orange), TCR constant domain C_β_ (yellow), TCR variable domain V_α_ (cyan), and TCR variable domain V_β_ (purple). Right panel top: Surface representation of the (partially overlapping) binding pockets A (blue), B (yellow), C (orange), D (cyan), E (red), and F (green). Right panel bottom: Licorice representation of the peptide consisting of nine residues (position 1: Glu, blue; position 9: Tyr, green) with their N- and C-termini buried in the binding pockets A (blue) and F (green), respectively. The figures were prepared using VMD version 1.9.3 [50] (University of Illinois, Urbana-Champaign, IL, USA)

The earlier mentioned studies of various TCR-pMHC systems provide valuable contributions to our current understanding of the adaptive immune response. In a previous work [18], we studied the TCR-pMHC-I system LC13-HLA-B*44:05-pEEYLQAFTY (see Fig. 1) and evaluated flexibility properties of the pMHC protein upon TCR ligation using MD simulations with a total length of 8 µs. This system is of particular interest as the LC13 TCR is selected for recognition of self-HLA-B*08:01 bound to the Epstein-Barr viral peptide FLRGRAYGL, but alloreacts with HLA-B*44:05 bound to EEYLQAFTY [5].

In the present study, we extended our previous findings regarding the pMHC-TCR system LC13-HLA-B*44:05-pEEYLQAFTY by adding additional analyses and considering further properties. In particular, we examined differences in the intramolecular flexibility of the free and the TCR-bound pMHC-I system with emphasis on the MHC binding groove as well as the antigenic peptide, which are involved in the TCR-pMHC complex assembly. We used the root mean square fluctuations (RMSFs) of individual residues of the MHC α1- and α2-helix as well as of the peptide to quantify the flexibility and to characterize relative movements of intramolecular domains in the free and the bound state. Furthermore, the radius of gyration (*R*_g_) of the MHC α1-helix, the MHC α2-helix, both MHC α-helices and the peptide was computed to characterize structural differences of MHC domains between the free and the bound state. To measure the compactness of the binding groove, the time-evolution of the inter-domain distance between the MHC α1- and α2-helix was evaluated for the free and the bound state. Additionally, the total number of H-bonds as well as the residues involved in the formation of the H-bonds were analyzed for the free and the bound state in order to get an understanding of the intermolecular pMHC interaction through hydrogen bonding. As molecular flexibility is directly related to conformational entropy, we estimated changes in conformational entropy of the binding groove upon TCR ligation. Finally, we computed the solvent accessible surface area (SASA), which is another useful descriptor in the energetic analysis of protein–ligand associations.

Our results contribute to a more detailed view on flexibility properties of the investigated free and TCR-bound pMHC-I system due to the long simulation time of 8 µs.

## Results

### RMSD of the free pMHC molecule and the pMHC-TCR complex

**Fig. 2:**
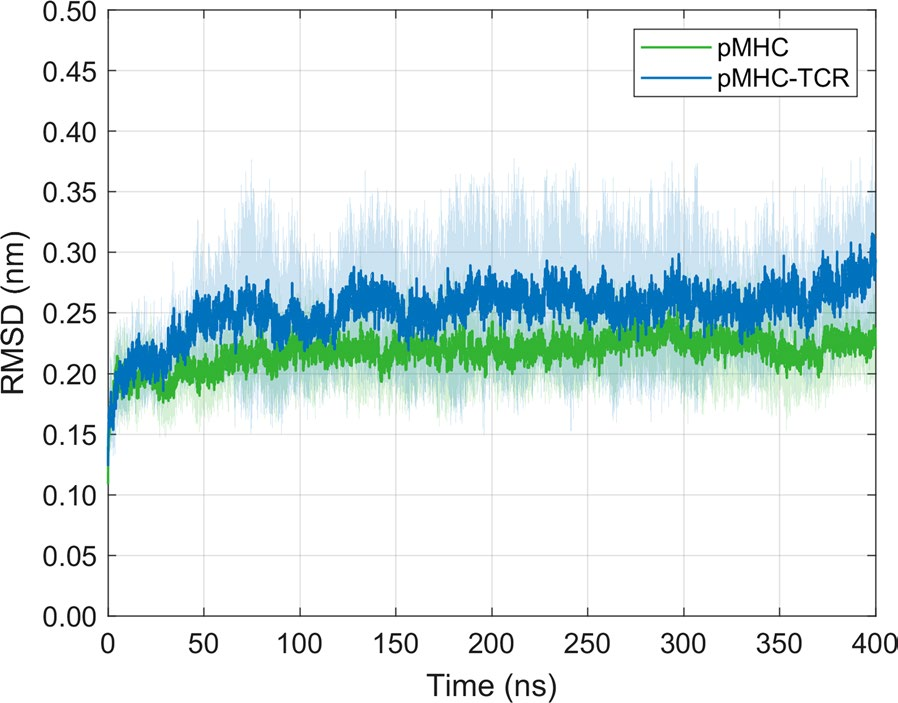
Time course of C_α_ RMSD-values (mean ± SD) for ten independent MD simulations of the free pMHC molecule and of the whole pMHC-TCR complex

The 3KPS pMHC-TCR protein represents a fairly complex system for MD studies, and one is well advised to examine the degree of equilibration prior to further analysis steps. Figure 2 shows the mean ± SD of C_α_ RMSDs calculated from 10 independent 400 ns simulations each for the free pMHC molecule and the whole 3KPS system. In the free pMHC system, after approximately 50 ns, the mean RMSD levels off and eventually reaches a plateau, indicating an approximately stationary probability distribution for the states of the system sampled by the MD simulations. In the pMHC-TCR complex, however, the mean RMSD increases after around 350 ns. This is caused by the RMSD courses of individual runs (see Additional file 1: Figs. S1, S2), mainly due to the movements of the MHC α3 and β2-microglobulin domains in the respective runs.

### Radius of gyration

**Fig. 3:**
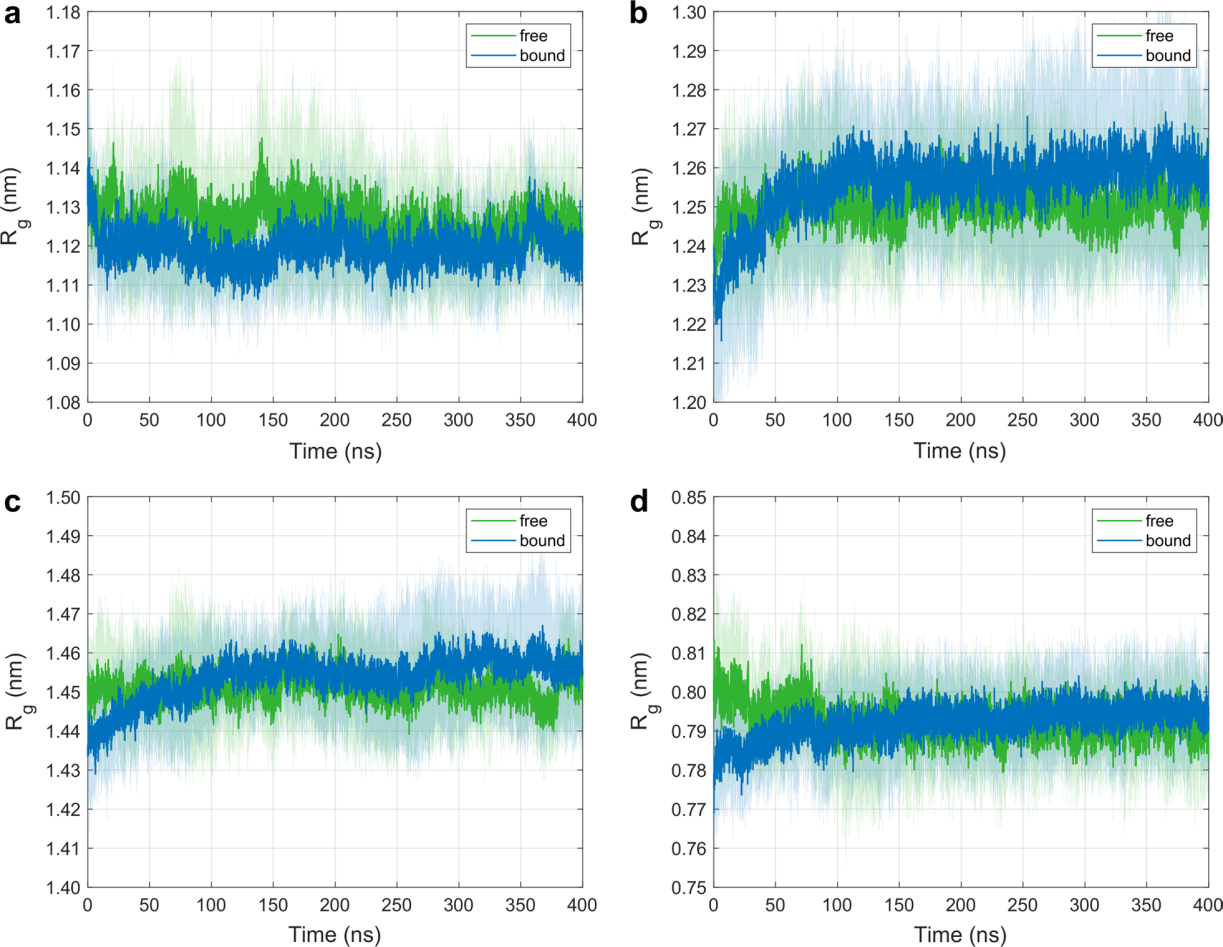
Time course of the radius of gyration *R*_g_ of C_α_ atoms (mean ± SD) for ten independent MD simulations of 400 ns each for the free and the TCR-bound state: **a** MHC α1-helix. **b** MHC α2-helix. **c** Both MHC α-helices. **d** MHC peptide

We employed the *R*_g_-descriptor to measure differences in the compactness of MHC-domains between the free and the bound state. Figure 3 displays time courses of *R*_g_-values (mean ± SD) as obtained from 10 independent 400 ns MD simulations for the free and the TCR-bound pMHC molecule. While the compactness of the α1-helix is increased in the bound state (Fig. 3a), the α2-helix shows a reversed behavior (Fig. 3b). For the MHC-peptide (Fig. 3d), *R*_g_-values are systematically elevated upon complex formation. The decreased compactness of the α2-helix upon TCR binding outweighs the opposite trend of the α1-helix, so that the two joint helices (Fig. 3c) and the peptide show a similar behavior. Even though these differences are small, they remain manifest after an equilibration phase during the first 100 ns of the simulations.

### Inter-domain distance between MHC α-helices

**Fig. 4:**
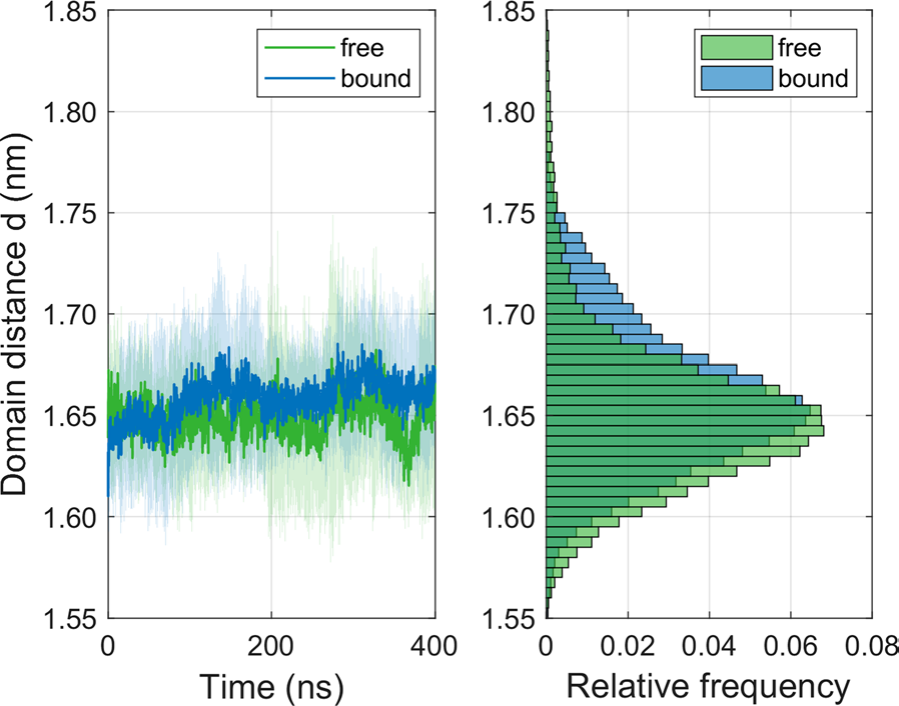
Time course and histogram of the inter-domain distance *d* (mean ± SD) between the α1- and α2-helix of the MHC for the free and the TCR-bound state as calculated from ten 400 ns simulations each

The proper presentation of the peptide to a TCR by the MHC binding groove is central to a successful initiation of an immune response. We therefore evaluated the time evolution of the inter-domain distance *d* between the α1- and α2-helix of the MHC binding groove in the free and the TCR-bound state, see Fig. 4. After a cross-over at approximately 50 ns, the time averaged distance between the helices only shows slight differences between the free and the bound state: < *d*_free_ >  = 1.65 nm, < *d*_bound_ >  = 1.66 nm, where < … > denotes the time average over the pooled 10 trajectories. After an initial equilibration of approximately 50 ns duration, mean distances in the bound state are systematically larger than in the free state.

### Root mean square fluctuations in the pMHC molecule

**Fig. 5:**
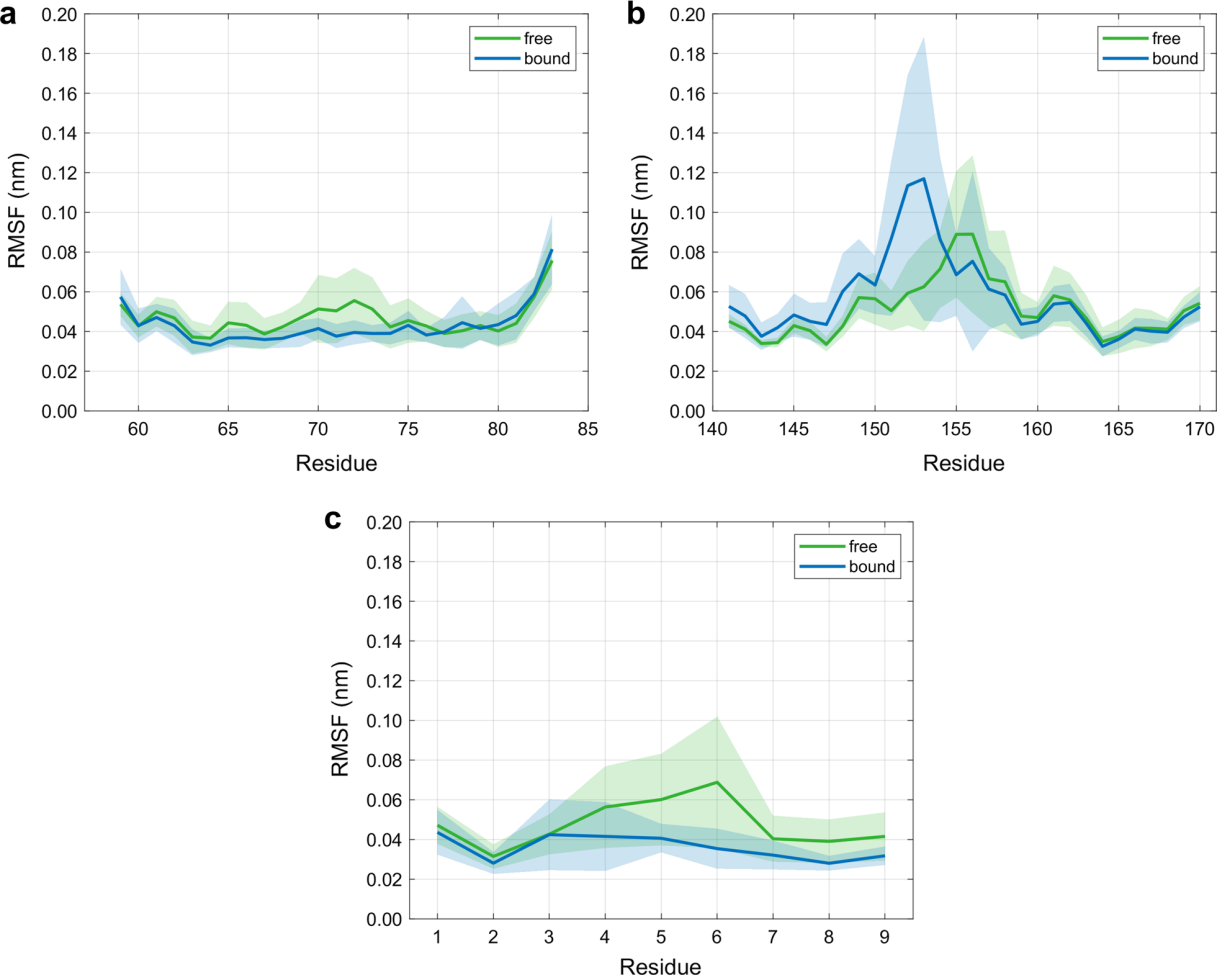
RMSF-values of residue C_α_ atoms (mean ± SD) for 10 independent MD simulations of 400 ns each for the free and the TCR-bound state: **a** MHC α1-helix. **b** MHC α2-helix. **c** MHC peptide. The first 50 ns of the individual simulations were excluded from the analysis

We evaluated C_α_ RMSF-values of individual residues of the MHC α1- and α2-helix as well as of the peptide, where the first 50 ns of individual simulations were excluded from the analysis. RMSF-values are generally decreased upon binding with the TCR or at most remain unaffected, with the exception of the MHC α2-helix, see Fig. 5. Most striking differences between free and bound RMSF-values were observed for residues at positions 4–6 (Leu, Gln, Ala) of the peptide as well as residues at positions 151–153 (Arg, Val, Ala) within the α2-helix and less pronounced changes were observed within the α1-helix at positions 70–73 (Asn, Thr, Gln, Thr). N- and C-terminal positions (residues at positions 1 and 9, respectively) of the peptide remain comparably stable upon TCR-binding with respect to the RMSF.

### H-Bonds of the peptide with the MHC binding groove

**Fig. 6:**
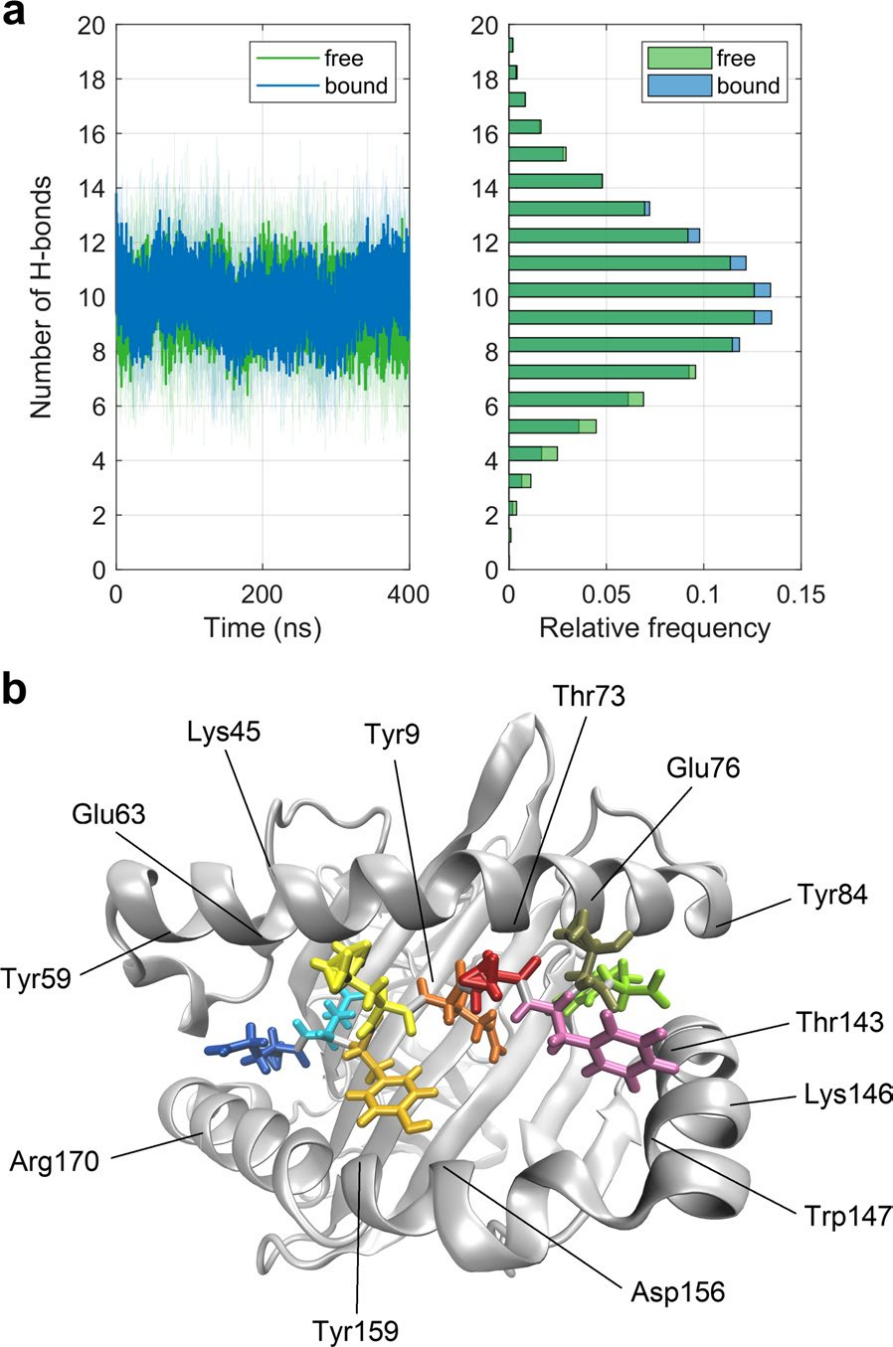
H-bonds between the peptide and the MHC binding-groove. **a** Time course and histogram of the total number of H-bonds (mean ± SD) calculated from 10 pooled 400 ns MD simulations each for the free and the bound state. **b** Cartoon representation of the MHC binding groove with lateral α1- and α2-helices and the β-sheet (grey) together with the peptide (shown as licorice) consisting of nine residues (position 1: Glu, blue; position 9: Tyr, green). Residues with H-bonds of a relative occurrence ≥ 25% in either bound, free or both states are labelled. The figure was prepared using VMD version 1.9.3 [50]

Figure 6a displays the time course of the total number of H-bonds (mean ± SD) between the peptide and the MHC binding groove, i.e., the lateral α-helices and the β-sheet underneath, as obtained from 10 independent MD simulations each for the free and the bound state. After an equilibration phase of 50 ns, the dynamics of the mean total number of H-bonds virtually does not change with time, nor do the differences between the free and the bound states with respect to their mean values: 9.7 ± 0.2 (free) versus 9.8 ± 0.3 (bound). There are systematic differences between the free and the bound state in the individual simulations (data not shown), albeit in mutually opposite directions, i.e., approximately half of the simulations show an increased, while the other half show a decreased total number of H-bonds in the bound state. Figure 6b shows a cartoon representation of the MHC binding groove, where residues with H-bonds of a relative occurrence ≥ 25% (see the following paragraph) in either the bound, the free or both states are labelled with their respective 3-letter codes.

**Fig. 7:**
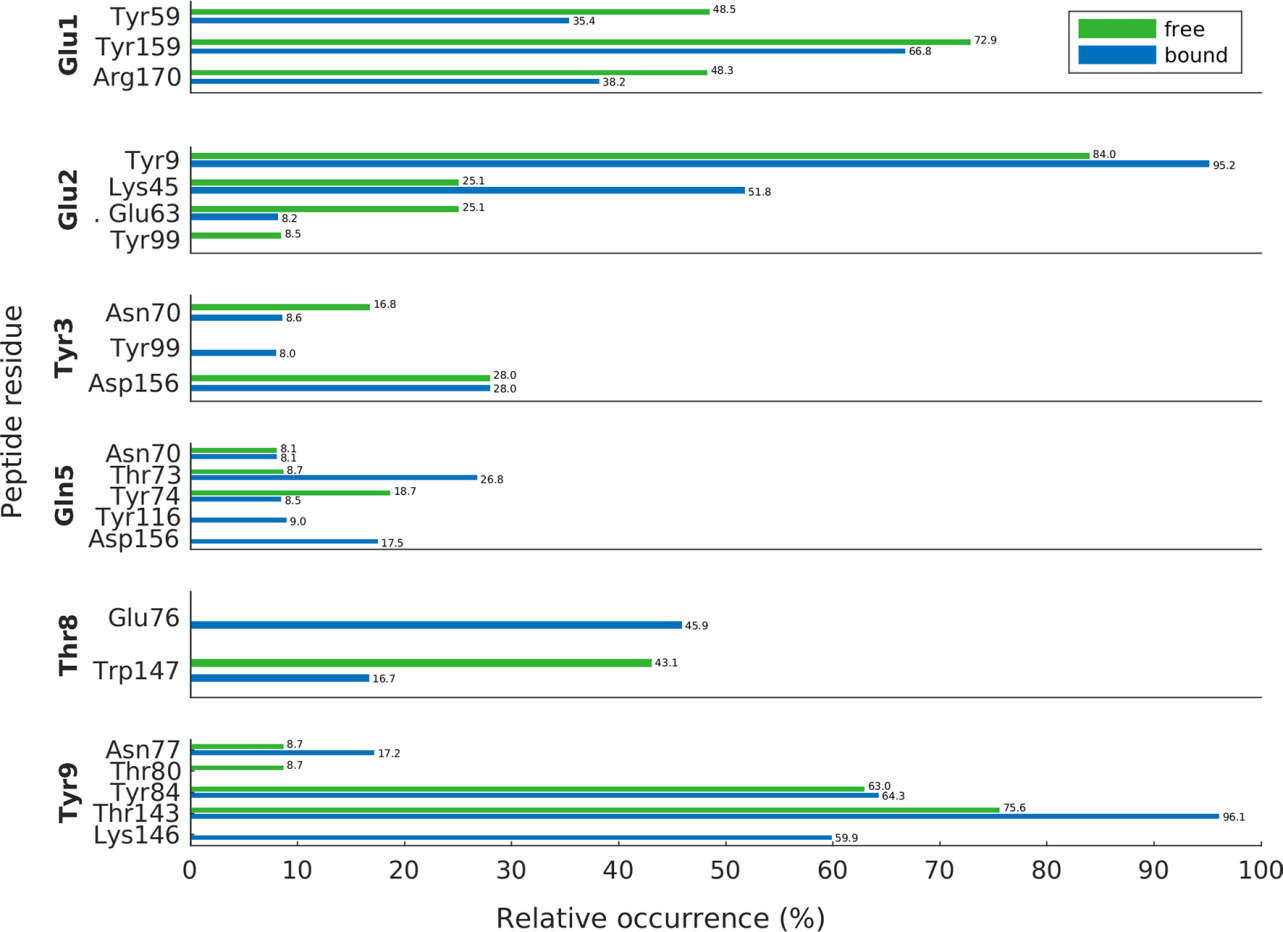
Relative occurrences of H-bonds between the residues of the peptide and the binding groove (i.e., average percentage of H-bonds weighted by the number of their appearance in the 10 individual runs each for the free and the bound state, irrespective of donors/acceptors located in the main or the side chain)

In addition to the progression of the total number of H-bonds between the peptide and the MHC binding groove, we analyzed residues engaged in forming the respective interactions, excluding the first 50 ns of the simulations. Figure 7 shows residues of the peptide and the binding groove, which are involved in H-bond interactions that were present more than 80% of the total simulation time in at least one of the 20 MD runs. Displayed are relative occurrences (i.e., average percentages of H-bonds weighted by the number of their appearances within the 10 individual runs each for the free and the bound state, irrespective of donors/acceptors located in the main or the side chain). Figure 7 demonstrates larger relative occurrences of H-bonds at the N-terminus (Glu1) of the peptide in the free state and larger relative occurrences of H-bonds at the C-terminus (Tyr9) of the peptide in the bound state. While many H-bonds were highly conserved during TCR ligation (e.g., Tyr3-Asp156, Tyr9-Tyr84), some others appeared only in the free state (e.g., Glu2-Tyr99, Tyr9-Thr80) or only in the bound state (e.g., Tyr3-Tyr99, Thr8-Glu76). Also, the comparably short presence of H-bonds involving peptide residue Ala6 is consistent with its large positional fluctuations in the free state, see Fig. 5c for the respective RMSF-values.

### Configurational entropy of the binding groove

**Fig. 8:**
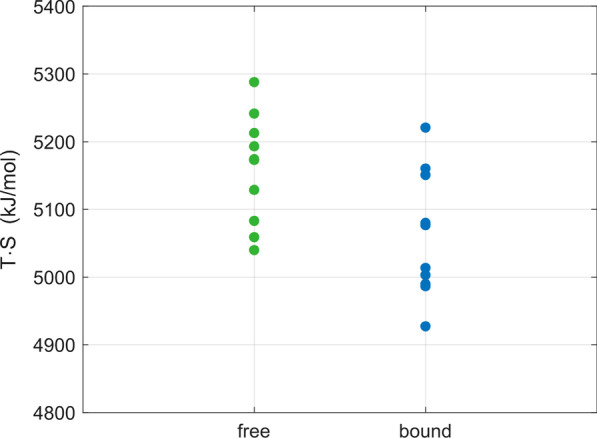
Dot plot of entropy estimates for the pMHC binding groove for each of the 10 runs of the free and the TCR-bound pMHC molecule, where the first 50 ns of the simulations were excluded from the analysis. Mean ± SD of entropic energy values *T·S* at *T* = 310 K: 5060.91 ± 93.16 kJ/mol (bound) *versus* 5159.33 ± 80.84 kJ/mol (free). Difference of mean values: *T*Δ*S* =  − 98.43 kJ/mol. Standard error of *T*Δ*S*: 39.0 kJ/mol. The difference *T*Δ*S* of respective mean values was statistically significant (two-sided *t*-test for independent samples, *p* = 0.021)

Figure 8 displays estimates of configurational entropies of the pMHC binding groove for each of the 10 runs for the free and the TCR-bound pMHC molecule, where the first 50 ns of the simulations were excluded from the analysis. Although we observed an overlapping range of entropic energy values *T·S* of the free and the bound states at *T* = 310 K, the difference *T*Δ*S* =  − 98.43 kJ/mol between the respective mean values of entropic energies (5060.91 ± 93.16 kJ/mol bound *versus* 5159.33 ± 80.84 kJ/mol free, mean ± SD) is statistically significant (two-sided *t*-test for independent samples, *p* = 0.021) with a standard error of the difference *T*Δ*S* of the mean values of 39.0 kJ/mol.

## Discussion

We performed a series of long-term MD simulations to analyze conformational and flexibility properties of a pMHC class I molecule upon TCR ligation. In particular, we conducted ten independent 400 ns runs each for an unbound and TCR-bound pMHC system, summing up to a total simulation time of 8 µs. We chose this design of the simulations, since conformational space has been reported of being more efficiently sampled by a series of relatively short and independent MD simulations than by a single long one [19].

### Backbone RMSD

Mean RMSD-values were adequately relaxed to equilibrium after an initial relaxation time of approximately 50 ns, suggesting that the simulations had generated stable trajectories, thus providing a sound basis for further analysis. Therefore, we removed the first 50 ns in the calculation of RMSF-values, in the analysis of H-bonds with VMD, and in the calculation of conformational entropies of the pMHC complex.

### Radius of gyration

To characterize the compactness of a structure, we evaluated the radius of gyration *R*_g_ of the MHC α1-helix, the MHC α2-helix, both MHC α-helices, and the peptide. We observed that relaxation times for equilibration were quite different both with respect to the domains considered (whole complex and, e.g., the peptide) and with respect to the descriptor (RMSD and *R*_g_), see Figs. 2 and 3. The different trend in *R*_g_-values of the α1-helix and the α2-helix upon binding might be due to inherent structural variations between the two helices: While the α1-helix constitutes a pure α-helix along all of its residues, the α2-helix is composed of three parts [20], joined by coils at residues 151 (Arg) and 162 (Gly), see Fig. 1.

### Inter-domain distance between MHC α-helices

Inter-domain distances *d* between the α1- and α2-helices are slightly elevated in the bound state after an initial equilibration phase of approximately 50 ns, where we observed a cross-over of the respective time courses, see Fig. 4. This behavior is consistent with the time course of *R*_g_-values for both α-helices, see Fig. 3c.

### Flexibilty within the pMHC molecule

**Fig. 9:**
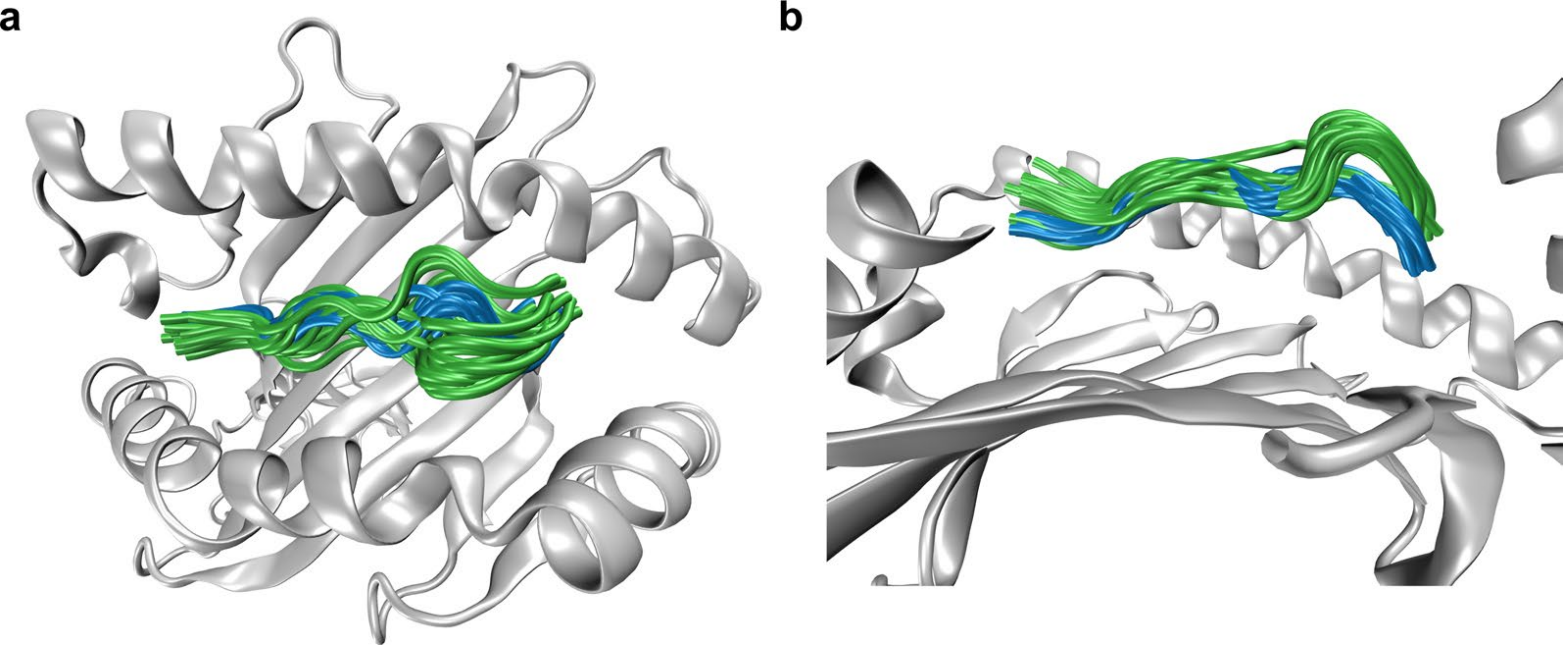
Snapshots illustrating peptide movements. **a** Cartoon representation of overlaid snapshots of MHC peptides at every 40 ns from representative 400 ns runs of the free (green) and the bound (blue) states to illustrate the extent of peptide movements. **b** Side view with parts of the α2-helix removed for better visibility of the peptide snapshots. The figures were prepared using VMD version 1.9.3 [50]

Flexibility properties of the peptide and the MHC are important for TCR ligation [6, 21, 22]. Changes in flexibility of pMHC domains (peptide and α-helices) upon TCR binding were characterized by C_α_ RMSF-values of the respective residues, see Fig. 5. In addition, Fig. 9 illustrates the extent of movements of the peptide in two representative 400 ns runs of the free and of the bound state. In line with previous studies [23, 24], we observed a reduced dynamic flexibility upon TCR ligation within the central residues of the peptide and less pronounced also at the MHC α1-helix. As the authors in [7] have pointed out, this reduced conformational heterogeneity upon binding leads to increased entropic costs for binding and to a weakened affinity. Changes of RMSF-values upon TCR ligation, in particular within the peptide and the α2-helix, reflect a certain degree of adaptation of the pMHC complex to the TCR, thus pointing to an induced fit binding model rather than supporting the conformational selection paradigm [25].

### H-Bonds between the peptide and the MHC binding groove

H-bonds between the peptide and the MHC binding groove (i.e., the lateral α-helices and the subjacent β-sheet) anchor the peptide in the MHC and thus guarantee appropriate presentation to the TCR interface. The RMSF-values demonstrate that the N- and C-termini of the peptide (residue positions 1 and 9) are much less affected by TCR-binding than its central residues (see Fig. 5) as these termini act as anchor sites through a network of hydrogen bonds which stabilize the peptide-binding domain [20]. In agreement with other studies [26], most H-bonds of the terminal residues of the peptide were retained both in the free and in the bound state, see Fig. 7. H-bonds between peptide Glu1 and α2-helix Tyr159, peptide Glu2 and α2-helix Tyr9 as well as between peptide Tyr9 und α2-helix Thr143 were present with the highest relative occurrences (Glu1-Tyr159: 72.9% free, 66.8% bound; Glu2-Tyr9: 84.0% free, 95.2% bound; Tyr9-Thr143: 75.6% free, 96.1% bound), suggesting an adequate anchoring of the peptide ends in their respective binding pockets A and F [1, 16], see also Fig. 1.

### Conformational entropy of the MHC binding groove

TCR recognition of a pMHC complex is guided by the binding affinity of the TCR, which is directly related to the change in Gibbs free energy Δ*G*_bind_ upon binding. Δ*G*_bind_ represents the difference of an enthalpic (Δ*H*_bind_) and an entropic (*T*Δ*S*_bind_) term, Δ*G*_bind_ = Δ*H*_bind_ − *T*Δ*S*_bind_. Therefore, configurational entropy *S*, which is an essential contribution to Δ*S*_bind_, can play an important role in modulating free energy changes during the association of pMHC molecules with a TCR, i.e., during the transition from a high entropy (disordered) unbound state to a low entropy (ordered) bound state [27, 28].

Thermal motions on a picosecond to nanosecond timescale are universal in protein dynamics and are believed to be directly related to protein conformational entropy. Motions on these timescales can be directly studied with nuclear magnetic resonance (NMR) experiments and can also be captured in MD simulations with realistic force fields and adequate sampling techniques. Although absolute entropies depend on the models of molecular motions, relative entropies were reported to be model independent [28]. To estimate conformational entropy changes of the MHC binding groove upon TCR ligation, we used the quasi-harmonic approximation developed by Schlitter [29], which is based on a previous work of Karplus and Kushick [30] on entropy estimation from the covariance matrix **σ** of atomic coordinate fluctuations.

In the 3KPS system, we observed a value of *T*Δ*S* =  − 98.43 kJ/mol for the mean entropic energy change of the peptide-binding groove upon TCR ligation. This value is consistent with results reported by Narzi et al. [14], who estimated conformational entropies of the binding groove of MHC class I proteins in complex with viral and self-peptides from MD simulations. On the other hand, our observed decrease in configurational entropy is also consistent with the reduced flexibility of the α1-helix and parts of the α2-helix upon binding, see Fig. 5.

### Solvent accessible surface area of the MHC binding groove

**Fig. 10:**
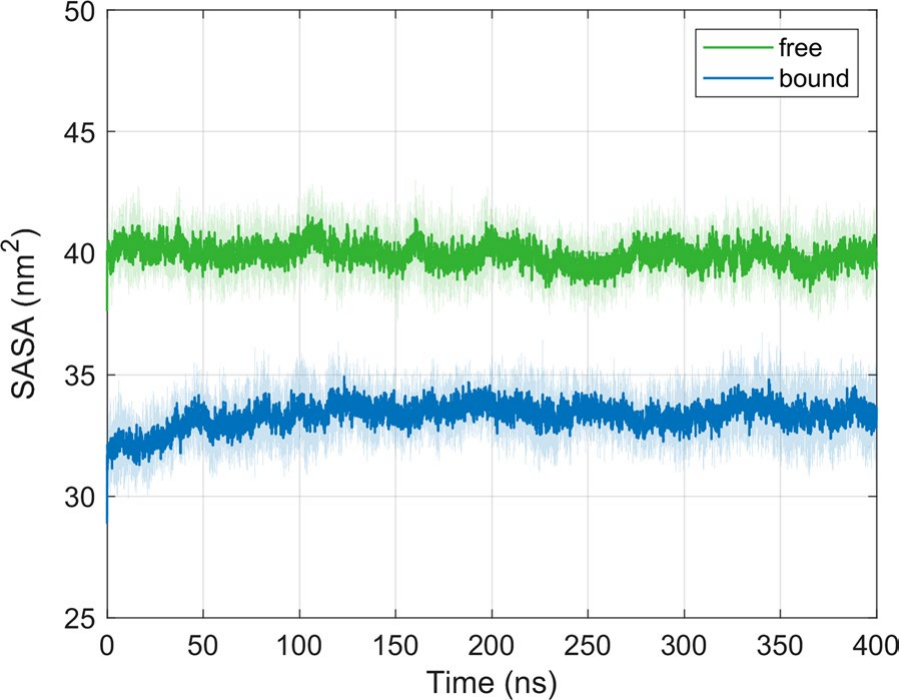
Time course of the solvent accessible surface area SASA (mean ± SD) of the MHC binding groove for the free and the TCR-bound state as calculated from ten 400 ns simulations each

The solvent accessible surface area (SASA) constitutes another useful descriptor in the energetic analysis of protein–ligand associations, e.g., in estimating solvation free energy changes. SASA values were calculated using the GROMACS tool *sasa* [31]. Figure 10 shows the time course of SASA values (mean ± SD) of the MHC binding groove in the free and the TCR-bound state as calculated from ten independent simulations of 400 ns each. After an initial transient phase of about 50 ns in the bound state, the SASA values remained fairly stable both in the free and in the bound state. The decrease upon TCR ligation by about 7 nm^2^ with respect to the free state is consistent with the view of a detachment of water molecules from the TCR-pMHC interface upon binding [32].

### Limitations

The membrane environment of pMHC and TCR proteins has been identified as an essential part of the pMHC-TCR interaction and the T cell response [33]. Since the seminal study of Wan et al. [34] of a membrane-embedded TCR-pMHC-II complex, many research groups have investigated the influence of the membrane on structural and thermodynamic properties of pMHC-TCR systems, e.g., [26, 35, 36]. In particular, Bello et al. [36] performed 100 and 150 ns MD simulations of peptide-bound and peptide-free MHC-II molecules in aqueous and membrane-bound configurations and reported that the presence of the membrane might restrict the conformational flexibility of the peptide-binding groove. Furthermore, co-receptors and various signaling proteins are involved in the formation of the immunological synapse and should also be included in future MD studies of pMHC-TCR systems [21].

Despite known issues associated with the Berendsen-barostat [37], we chose this algorithm to render our results better comparable with previous studies, e.g., Wan et al. [34], Narzi et al. [14], Bello and Correa-Basurto [35]. Our total simulation time of 8 µs for both the bound and the free system is still too short to capture processes with time scales far beyond this limit, such as slow complex motions or the initiation of TCR-signaling. To overcome the limits of simulation time, various approaches have been reported in the literature, such as coarse-grained methods, elastic network models, or steered MD simulations [38–40].

## Conclusions

In the present study, we used a series of long-term MD simulations to examine dynamic features of various domains of the HLA-B*44:05-pEEYLQAFTY complex upon binding with an αβ LC13 TCR. As a consequence of TCR-binding, we observed a reduced dynamic flexibility in the central residues of the peptide and the MHC α1-helix, altered occurrences of H-bonds between the peptide and the MHC, a reduced conformational entropy of the peptide-binding groove, as well as a decreased SASA. Moreover, our results suggest a minimum simulation time of approximately 100 ns for biomolecules of comparable complexity to obtain meaningful results. Furthermore, due to the large variability of individual RMSD-values as depicted in Fig. 2, we suggest a reasonable number of replicas for each system (depending on the specific descriptors studied) to draw reliable conclusions from conventional all-atom MD simulations in explicit solvent.

## Methods

### Simulation systems

We employed two different sets of start configurations for the MD simulations: The first set was made up of the crystal structure of a LC13 αβ TCR, a nine-mer self-peptide (EEYLQAFTY) derived from the ATB binding cassette protein ABCD3, and a MHC molecule of the HLA-B*44:05 type (PDB-ID: 3KPS) [5], see Fig. 1. This system holds the advantage of being available at a high spatial resolution of 2.7 Å. In the second set, MD simulations were started from unbound pMHC-molecules assembled from the crystal structure of the original 3KPS complex.

### Molecular dynamics simulations

We conducted all-atom MD simulations in explicit water with GROMACS 5.1.1 [41], using the Amber99sb-ildn force field [42] and a rhombic dodecahedral box with 2 nm minimum distance between each protein and the box boundaries. The 3KPS complex comprised 396,833 atoms, representing 826 protein residues and 127,634 solvent molecules. To reach a physiological salt concentration of 0.15 mol/L, the proteins were solvated in SPC water [43] and neutralized by replacing 748 solvent molecules with 386 Na^+^ and 362 Cl^−^ ions.

After energetically minimizing the systems using the method of steepest-descent with 10,000 steps, equilibration runs of 100 ps in length were performed at NVT conditions with the temperature set to 310 K by means of a Berendsen-thermostat with a time constant of 0.1 ps and position restraint MD. The following equilibration runs in NPT ensembles were conducted under the control of a Berendsen-barostat set to 1 bar with a time constant of 1.0 ps.

Production runs of 400 ns each were performed using the LINCS algorithm [44] for all bonds and virtual sites for hydrogen atoms, thus allowing for a time-step of 4 fs [41]. A cut-off of 1.47 nm was used for van der Waals interactions and the particle-mesh Ewald (PME) algorithm [45] was applied to calculate long-range electrostatic Coulomb interactions with a cut-off of 1.4 nm. Temperature coupling was conducted with the velocity-rescaling algorithm [46] at a temperature of 310 K. Isotropic pressure coupling at 1 bar was performed using the Berendsen algorithm [47]. Atom coordinates were saved every 40 ps to a trajectory file to yield 10,000 frames for each run. We carried out 20 independent MD simulations employing different initial velocities. Ten runs were performed for the whole complex and another 10 runs for the unbound pMHC molecule, resulting in a total simulation time of 8 µs.

### Structural analysis

Before analyzing the trajectories, translational and rotational motions of the proteins relative to their respective first frames (i.e., the equilibrated structures) were removed by means of the *trjconf* tool supplied with GROMACS.

To assess stability and adequate equilibration of the simulated systems, we computed the RMSD(*t*) (root mean square deviation) for each time-frame *t* with respect to the first frame of each trajectory with the GROMACS *rms* tool, considering only C_α_ atoms of the protein backbone:

$${\text{RMSD}}\left( t \right) = \sqrt {\frac{1}{N}\mathop \sum \limits_{i = 1}^{N} \left| {{\textbf{r}}_{i} \left( t \right) - {\varvec{r}}_{i}^{{{\text{ref}}}} } \right|^{2} }$$

where ***r***_*i*_(*t*) is the position of atom *i* at time *t*, *N* is the total number of C_α_ atoms within the protein backbone, and ${\varvec{r}}_{i}^{{{\text{ref}}}}$ is the position of atom *i* in the selected reference structure (the first frame of each trajectory in our case). The descriptor RMSD(*t*) thus represents the square root of averaged squared distances between the atom positions at time *t* and the positions in the reference structure, thereby rating molecular deformations at time *t* with respect to the reference positions at time zero.

To characterize the flexibility of individual atoms *i* or atom groups (e.g., residues), we evaluated the root mean square fluctuation RMSF(*i*) with the *rmsf* tool of GROMACS:

$${\text{RMSF}}\left( i \right) = \sqrt {\frac{1}{T}\mathop \sum \limits_{{t_{j} = 1}}^{T} \left| {{\varvec{r}}_{i} \left( {t_{j} } \right) - \left\langle {{\varvec{r}}_{i} } \right\rangle } \right|^{2} }$$

where *T* is the number of time steps over which the average is computed, *t*_*j*_ is the time step of frame *j* within the respective trajectory, and < ***r***_*i*_ > is the time-averaged position of atom *i*. Contrary to the RMSD(*t*), which represents an average over all atoms for a specific time *t*, the RMSF(*i*) is an average over time for a specific atom *i*. As such, the RMSF(*i*) constitutes a statistical measure of the deviation between the positions of an atom *i* during a simulation and its time-averaged position < ***r***_*i*_ >.

Molecular flexibility is directly related to conformational entropy, since increased molecular flexibility populates more states and thus entails higher entropy values [7]. To estimate changes in conformational entropy *S* of the binding groove upon TCR ligation, we employed the quasi-harmonic approximation developed by Schlitter [29, 48] as supplied with the GROMACS tools *covar* and *anaeig*:

$$S = \frac{k}{2}\ln \det \left( {\frac{{kTe^{2} }}{{\hbar^{2} }}{\mathbf{M}}{ }{{\varvec{\upsigma}}} + {\mathbf{I}}} \right)$$

where **M** and **I** are the mass matrix and the identity matrix, respectively, and *e* is Euler’s number. **σ** is the covariance matrix of the coordinate fluctuations computed from the MD trajectories,

$$\sigma_{ij} = \left( {x_{i} - \left\langle {x_{i} } \right\rangle } \right)\left( {x_{j} - \left\langle {x_{j} } \right\rangle } \right)$$

where *x*_1_,…, *x*_3*N*_ are the Cartesian coordinates of the *N* atom system and < … > stands for the time average. *k*, *T*, and *ħ* are Boltzmann’s constant, absolute temperature, and *ħ* = *h*/(*2π*), where *h* denotes Planck’s constant.

To describe relative movements of the α1- and α2-helices in the MHC molecule, we calculated the distance *d* between the geometric centers of these domains based on the C_α_ atoms of the protein backbones.

To probe differences in compactness between free and bound states, we also analyzed the radius of gyration *R*_g_ of C_α_ atoms in selected domains with the *gyrate* tool of GROMACS:

$$R_{{\text{g}}} = \sqrt {\frac{{\mathop \sum \nolimits_{i = 1}^{N} \left| {{\mathbf{r}}_{i} \left( t \right)} \right|^{2} m_{i} }}{{\mathop \sum \nolimits_{i = 1}^{N} m_{i} }}}$$

Here, *m*_*i*_ means the mass of atom *i* and ***r***_*i*_ denotes the position of atom *i* with respect to the center of mass of the molecule or a domain within the molecule. *R*_g_ is defined as the mass-weighted root mean square distance of a group of atoms from their common center of mass [49] and thus serves as a measure of the compactness of a structure.

Finally, we evaluated the time course of H-bonds between the peptide and the MHC binding groove using the *hbond* tool available within GROMACS. To examine the residues participating in the formation of H-bonds, we employed the *Hydrogen bonds* analysis tool of the VMD software [50]. H-bonds between donor- and acceptor-atoms were identified by means of the following geometrical criteria: (a) Donor–acceptor distance ≤ 3.5 Å, (b) hydrogen-donor–acceptor angle ≤ 30°. The donor–acceptor distance of 3.5 Å stems from the first minimum of the radial distribution function of SPC water.

## Supplementary Information

**Additional file 1.** Supplementary Figures.

## Data Availability

Data for MD simulations of the 3KPS system were downloaded from the PDB protein data bank: https://www.rcsb.org/structure/3KPS. Datasets generated by MD simulations are available from the corresponding author on reasonable request.

## Abbreviations

MD: Molecular dynamics
MHC: Major histocompatibility complex
pMHC: Peptide-MHC complex
RMSD: Root mean square deviation
RMSF: Root mean square fluctuation
*S*: Configurational entropy in kJ/(mol K)
SASA: Solvent accessible surface area
SD: Standard deviation
*T*: Absolute temperature in Kelvin (K)
TCR: T cell receptor
