# Supplementary material for: Conformational flexibility of a free and TCR-bound pMHC-I protein investigated by long-term molecular dynamics simulations
Source: BMC Immunol. 2022 Jul 29;23(Suppl 1):36. doi: 10.1186/s12865-022-00510-7 (PMC9335952; doi:10.1186/s12865-022-00510-7)
Supplement: Supplementary file 1 — Additional file 1. Supplementary Figures. [file 12865_2022_510_MOESM1_ESM.pdf]

Conformational flexibility of a free and TCR-bound pMHC-I protein investigated by long-term molecular dynamics simulations

Supplementary Material

Lisa Tomasiak[1], Rudolf Karch[1*] and Wolfgang Schreiner[1]

[1] Institute of Biosimulation and Bioinformatics, Center for Medical Statistics, Informatics and Intelligent Systems, Medical University of Vienna, Spitalgasse 23, 1090 Vienna, Austria

[*] To whom correspondence should be addressed

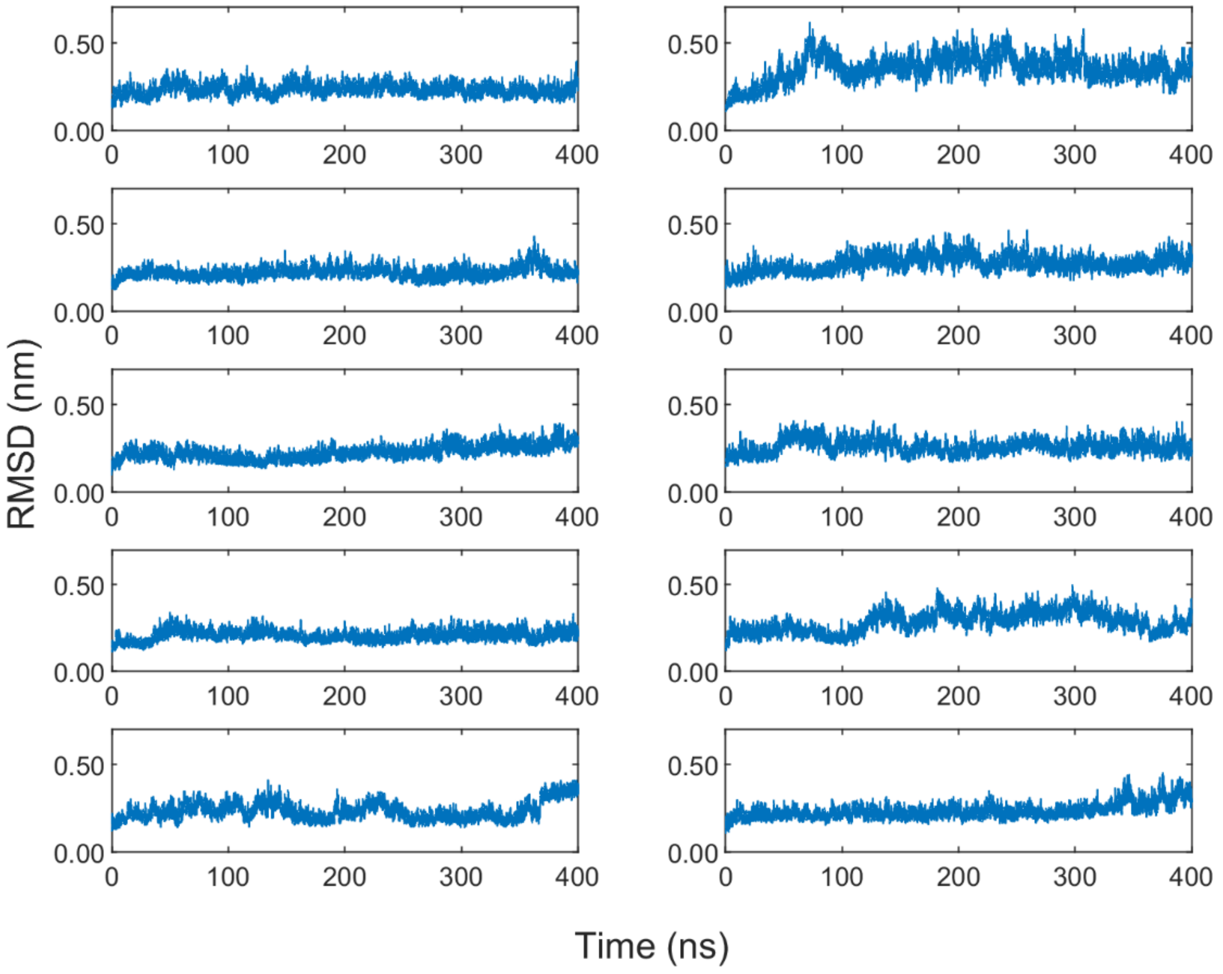

Fig. S1 Time courses of C_α RMSD-values of 10 independent MD simulations of the whole pMHC-TCR complex.

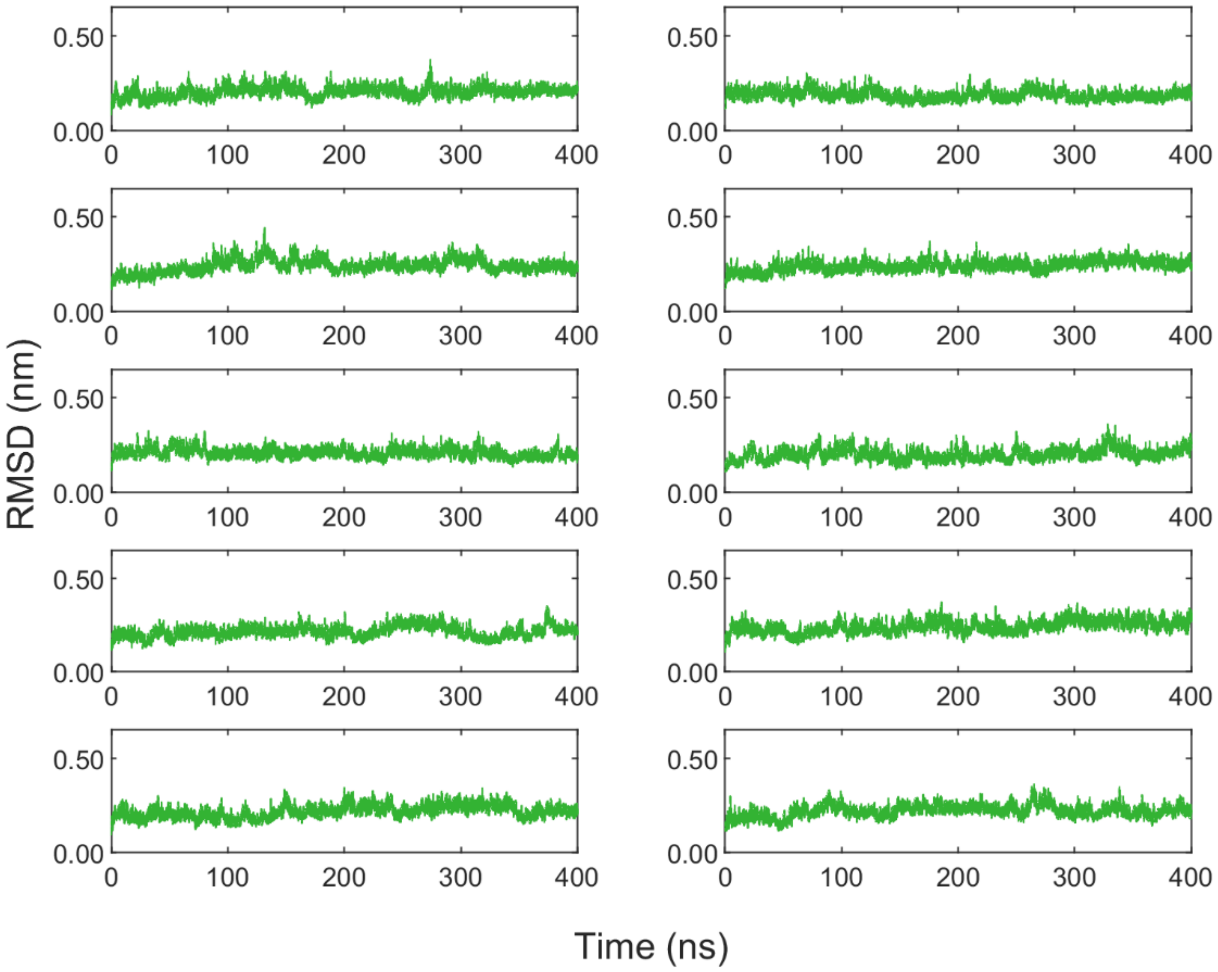

Fig. S2 Time courses of C_α RMSD-values of 10 independent MD simulations of the free pMHC system.
